# Implementation of a Modified Neonatal Early-onset Sepsis Calculator in Well-baby Nursery: a Quality Improvement Study

**DOI:** 10.1097/pq9.0000000000000330

**Published:** 2020-07-07

**Authors:** Michael Zayek, Jayalakshmi Bhat, Katie Bonner, Michelle Blake, Keith Peevy, Om Prakash Jha, Rashmi Gulati, Ramachandra Bhat

**Affiliations:** From the * Department of Pediatrics, Division of Neonatology, University of South Alabama, Mobile, Ala.; † Department of Pediatrics, University of South Alabama, Mobile, Ala.; ‡ University of South Alabama College of Medicine, Mobile, Ala.; § Department of Nursing, Children’s and Women’s Hospital, Mobile, Ala.

## Abstract

**Background:**

The use of sepsis risk scores (SRSs), calculated based on the neonatal early-onset sepsis (EOS) calculator, has been shown to limit the unwarranted sepsis evaluations and to reduce the empirical use of antibiotics in neonates.s

**Purpose:**

To reduce both the sepsis evaluation rate (SER) and antibiotic initiation rate (AIR) by 25% from baseline by incorporating conservative SRS cutoff values into the routine sepsis risk assessment of well-appearing neonates born at 34 weeks and older gestation.

**Methods:**

During a pre quality improvement (QI) period (June 2016–August 2016), a QI team calculated SRS on all newborn infants to determine safe SRS cutoff values. During the QI-study period (September 2016–November 2017), we implemented an EOS evaluation algorithm based on 2 SRS cutoff values, 0.05 (later increased to 0.1) for sepsis evaluation and 0.3 for the initiation of antibiotic therapy. Monthly SER and AIR were summarized and analyzed by using standard statistical tests and statistical process control charts. During the surveillance phase (January 2019–June 2019), we evaluated whether previously attained improvements in SER and AIR were sustained.

**Results:**

During the pre-QI period, the mean (±SD) of monthly SER and monthly AIR were 23.8% (±5.7%) and 6.2% (±0.4%), respectively. During the QI-study period, the mean (±SD) of monthly SER and monthly AIR decreased to 15% (±4.7%), *P* = 0.01, and 3.2% (±1.5%), *P* = 0.005, respectively. During the surveillance period, both outcome measures were comparable with the QI-study period.

**Conclusion:**

The implementation of a modified EOS calculator-based EOS algorithm using a conservative approach was successful in reducing antibiotic exposure and the need for blood work in well-appearing neonates.

## Abstract 2

Supplemental Digital Content is available in the text.

## INTRODUCTION

The overuse of antibiotics during the neonatal period is unjustified as early postnatal antibiotic exposure is associated with increased risks of asthma,^1^ obesity,^2^ allergic disorders,^3^ and diabetes mellitus^4^ at a later age. Also, unnecessary antibiotic use contributes to the emergence of antibiotic-resistant bacteria.^5^ As a result, the Center for Disease Control and Prevention (CDC) attempted to solve the problem of overuse of antibiotic therapy by publishing improvised evaluation guidelines for risk stratification of infants at risk for early-onset sepsis (EOS) in 2010.^6^ Despite these recommendations, approximately 5%–10%^7^ of healthy newborn infants remained unjustifiably exposed to antibiotics while the current incidence of EOS is only 0.3–0.6 per 1,000 live births.^6,7^ The ambiguity in applying the CDC 2010 EOS guidelines was the main barrier for achieving the minimization of antibiotic overuse, because under the CDC 2010 guidelines, the risk factors such as gestational age, maternal chorioamnionitis, duration of rupture of membrane, Group B streptococcus colonization status, and intrapartum antibiotic prophylaxis were defined as categorical variables. However, in reality, these risk factors were either continuous variables (highest maternal temperature, duration of ROM, and gestational age) and multilevel categorical variables (Group B streptococcus status and intrapartum prophylaxis).^8^ Also, the CDC 2010 EOS guidance lacked explicit instructions on the type of interventions, which led to the publication of the American Academy of Pediatrics (AAP) EOS guidelines^9^ in 2012 and various institutional algorithms.^10^

In 2010, researchers at Kaiser Permanente Northern California (KPNC) developed a web-based neonatal EOS calculator^11,12^ that computes a sepsis risk by utilizing the individual risk factors for sepsis and generates EOS risk estimates [sepsis risk scores (SRSs)] based on the postnatal clinical categorization of the infant. The algorithm also suggests a specific management pathway for each clinical status class.^12–14^ In recent years, several institutions have incorporated the EOS calculator into their practice after it was shown to successfully reduce the need for unnecessary sepsis evaluations and antibiotic initiations in a large population of infants.^14^ Also, the neonatal EOS calculator use potentially decreases the proportion of infants needlessly transferred to higher acuity levels of care and reduces maternal–infant separation^.^.^10,15^

Nevertheless, many other institutions remain hesitant to implement the neonatal EOS calculator because its safety is not well established. Although its use has lowered rates of empirical antibiotic use and the amount of blood sampling, its use has also led to a delayed initiation of antibiotic therapy in infants, many of whom would have otherwise been identified and treated early under CDC/AAP EOS guidelines.^14,16,17^ Besides, the fear of medicolegal liability when adapting to a new paradigm that has not been formally adopted by national authorities such as CDC and AAP can be a significant barrier to various administrative and medical professionals.^18^

To combat these concerns, we decided to undertake the implementation of the EOS calculator-based evaluation and management of EOS in neonates under multiple phases using multiple Plan-Do-Study-Act (PDSA) cycles. In the current paper, we describe the initial phase of the multiphase quality improvement (QI) initiative, wherein we used extremely conservative SRS cutoff values. In this phase, we undertook the implementation of the neonatal EOS calculator only in a population of well-appearing newborns. Our smart aims are to reduce both sepsis evaluation and antibiotic usage by 25% from baseline values by routinely implementing the EOS calculator into the care of all well-appearing newborn infants who are born at 34 weeks and older of gestational age and admitted to a level-1 newborn nursery.

## METHODS

### Setting

This study is a single-center QI initiative, which was approved by the institutional review board and was conducted at a level-1 newborn nursery, Children’s and Women’s Hospital, University of South Alabama, Mobile, Ala. The newborn nursery is an academic neonatal unit with a capacity of 45 beds and an average admission rate of 2,000 newborns per year, where well-appearing newborn infants born at 34 weeks and older’ gestation get care. A total of 7 neonatologists, 6 nurse practitioners, 45 registered nurses, and several pediatric residents provide care to these healthy neonates.

### Design

This QI initiative comprised 3 periods: pre-QI period (baseline), QI-study period, and a surveillance period.

#### Pre-QI period (baseline)

The pre-QI period extended from June 2016 through August 2016, during which we prospectively collected the data to provide the most accurate baseline data on sepsis evaluation and antibiotic utilization at our center. The QI team computed SRS using the web-based neonatal EOS calculator on each infant separately from the clinical team, using an institutional risk of 0.3 per 1,000 live births. During this period, we did not use the SRS for the management of individual infants. Still, it was used to determine the institutional cutoff values that can safely reduce antibiotic overusage without increasing the risk of delayed antibiotic initiation among infants with culture-proven EOS and clinical EOS. The evaluation and management of well-appearing newborn infants did not change from previous practices as physician-led decisions continued to follow the CDC 2010 and AAP 2012 EOS guidelines.

#### QI-study Period (QI Interventions)

The QI-study period consisted of 3 PDSA cycles, which extended from September 2016 through November 2017. During this period, we introduced QI interventions sequentially along with prospective data collection and evaluations of the outcome measures.

##### *PDSA Cycle 1*.

**Fig. 1.**
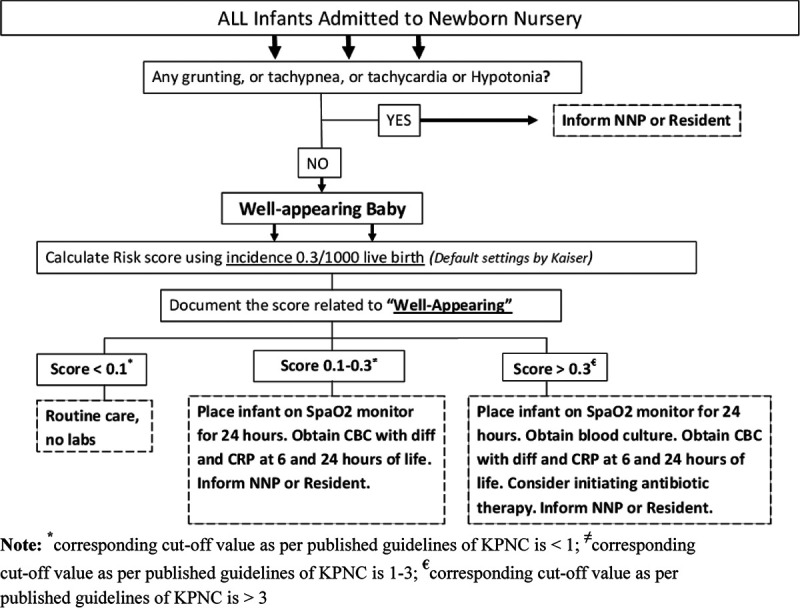
Final web-based EOS calculator-based EOS sepsis algorithm developed for our institution. * indicates corresponding cutoff value as per published guidelines of KPNC is <1; ≠ indicates corresponding cutoff value as per published guidelines of KPNC is 1–3; € indicates corresponding cutoff value as per published guidelines of KPNC is >3.

**Fig. 2.**
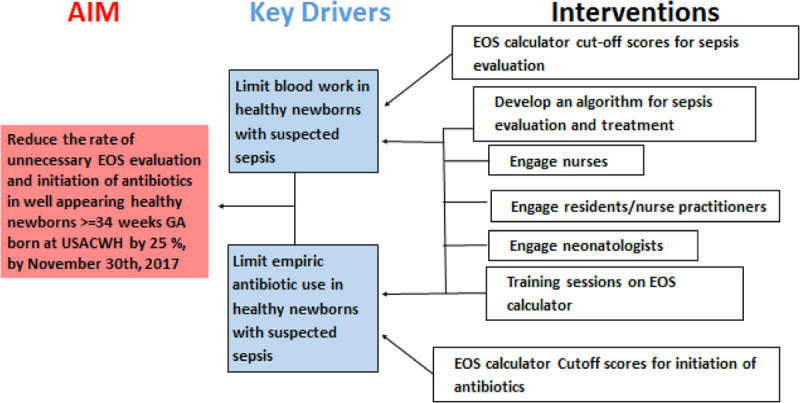
Key driver diagram.

We formed a multidisciplinary QI team in the newborn nursery, which consisted of 2 nurse practitioners, 2 nurse educators, a pediatric resident, 3 neonatologists, and 1 nurse manager. For the smooth and efficient implementation of the web-based neonatal EOS calculator, the QI team developed a simplified institutional algorithm (Fig. 1) to guide sepsis assessment and management. It was explicitly designed for well-appearing newborns and was based on 2 separate SRS cutoff values, a cutoff value of 0.05 for limited sepsis evaluation [complete blood count (CBC) and serial C-reactive protein (CRP) measurements] and a cutoff value of 0.3 for a full sepsis evaluation (including blood culture) and antibiotic therapy. By consensus, we chose these values after reviewing the SRS of all the well-appearing infants admitted to the newborn nursery during the pre-QI phase. Based on the available institutional data on SRS, we estimated that the use of a cutoff limit of 0.05 would have decreased the limited laboratory evaluations by approximately 50%. During this period, the QI team trained all nurses, nurse practitioners of the newborn nursery, and residents on the use of the web-based neonatal EOS calculator. Although these SRSs were calculated and incorporated into the electronic medical records along with all the variables used for calculation, they were not used to make management decisions. During this PDSA cycle, physician-led decisions continued to follow the CDC 2010 and AAP 2012 guidelines. We also developed a key-driver diagram and established outcome goals (Fig. 2).

##### *PDSA Cycle 2*.

Once the staff members demonstrated optimal proficiency in using the web-based EOS calculator and navigating through the algorithm to arrive at the management decision based on the obtained risk estimate scores, we initiated the routine use of the algorithm. Since the time it was implemented, management and evaluation decisions were based on the institutional algorithm with its specified cutoff scores.

##### *PDSA Cycle 3*.

Tasks, including active staff engagement in collecting all the variables required for the EOS risk calculation, along with the ongoing education of staff members, were continued. Review of the safety and efficacy data allowed for a consensus to be reached and to increase the cutoff value of 0.05 to 0.1 for initiating the limited sepsis evaluation. Education and feedback continued along with prospective data collection.

Overall, we have taken the following conservative steps for a cautious implementation of the EOS calculator-based EOS algorithm at our institution:

1) A multiphase approach in which we initially involved only the well-appearing infants (the current study).
2) The use of SRS cutoff values that were 10-fold lower than the published guidelines of KPNC (Fig. 1).
3) The use of CBC and serial CRP as biomarkers in addition to the clinical monitoring (Fig. 1).
4) Obtaining blood culture at the SRS cutoff value 3-fold lower than KPNC guidelines.

#### Surveillance Period

During this period, from January 2019 through June 2019, we retrospectively reviewed the sepsis evaluation and antibiotic utilization to assess the sustainability of previously achieved improvements in well-appearing newborn infants. We retrospectively collected the relevant sociodemographic, clinical, SRS data, and laboratory data on all well-appearing neonates, 34 weeks and older of gestation, admitted to the newborn nursery. The outcome measures were calculated monthly and plotted on statistical process control charts and compared with the QI study period’s data.

### Measurements

The outcome measures were monthly sepsis evaluation rates (SERs) and monthly antibiotic initiation rates (AIRs). We defined sepsis evaluation as either limited evaluation comprised CBC and serial CRP estimation or complete evaluation with added peripheral blood culture (before initiating the antibiotic therapy). Lumbar puncture was performed in only selected cases. For the process measures, we tracked the appropriate and consistent utilization of the web-based EOS calculator for making decisions about the evaluation and management of EOS in well-appearing neonates. We also monitored deviation rates from the sepsis evaluation protocol when interventions or treatments initiated were not justified by the institutional algorithm. For balancing measures, we monitored any delayed diagnosis, including readmission diagnosis within 7 days, of clinical sepsis or culture-proven sepsis.

### Data Source, Data Collection, and Data Analyses

We collected relevant maternal, perinatal, clinical, laboratory, and treatment data. The changes in outcome measures over time, SER and AIR, were analyzed by using conventional statistical methods, statistical process control charts, and run charts. The outcome data were reported at an aggregate level for the 3 periods and also for each PDSA cycles, and the mean ± SD of outcome measures were compared using the analysis of variance test. As there are multiple levels, we performed posthoc pairwise multiple comparisons to analyze the differences in the outcomes between different phases. We applied Bonferroni for the various comparisons and calculated Bonferroni adjusted *P* values and adjusted 95% confidence interval (CI) for the pairwise comparisons. To demonstrate the significant impact of the sequentially introduced interventions, we analyzed the outcome measures using the p-chart (statistical process control chart) method.^19^ The upper control limit (UCL) and lower control limit (LCL) were set at ±3 SD. We used the standard Montgomery rules to determine special cause variations.^20^ Centerline, UCL, and LCL were recalculated after the special-cause signal shift. Centerline and UCL and LCL were also recalculated for the surveillance period.

## RESULT

During the study period (pre-QI and QI-study period), a total of 3,164 infants were admitted to the newborn nursery. We excluded a total of 232 (7%) non well-appearing infants due to the presence of equivocal or cardiorespiratory symptoms needing neonatal intensive care transfer. We summarize the baseline characteristics of the included population in the table for the baseline period, each PDSA cycle, and the surveillance period of the study (**see Table, Supplementary Digital Content,** http://links.lww.com/PQ9/A204). During the baseline phase, the monthly SERs were higher and nonuniform with wide variations, ranging from 18.7% to 30% with a mean (±SD) of 23.8% (±5.7%). During the QI-study period (3 PDSA cycles included), the mean (±SD) of SER was 15% (±4.7%), which decreased significantly from the baseline SER (pre-QI period) [mean difference (95% CI) = −8.8% (−16.1%, −1.4%), *P* = 0.01]. During the baseline period, the monthly AIR was consistently high with minimal variations, with a range of 5.8%–6.6% and with a mean (±SD) of 6.2% (±0.4%). Overall, the mean (±SD) of monthly AIR of the QI-study period was 3.2% (±1.5%), which decreased significantly from the baseline monthly AIR [mean difference (95% CI) = −3.1%% (−5.3%, −1%), *P* = 0.005].

A total of 889 infants born at Children’s and Women’s Hospital born during the surveillance phase of the study were evaluated for inclusion. We excluded 89 infants that were non-well appearing (**see Table, Supplementary Digital Content,** http://links.lww.com/PQ9/A204). During the surveillance period, mean (±SD) monthly SER and monthly AIR were 11.3% (±4.4%) and 2.6% (±1.4%), respectively. Monthly SER and AIR were comparable with SER and AIR during the QI-study period [mean difference (95% CI) = −4.1% (−9.6%, 1.4%), *P* = 0.21] and −0.6% (−2.3%, 1.1%), *P* = 0.99, respectively]. Both SER and AIR (mean ± SD) during the surveillance period remained lower when compared to SER and AIR during the pre-QI baseline period [mean difference (95% CI) = −12.8% (−21.1%, −4.7%), *P* = 0.002 and −3.7% (−6.1%, −1.2%), *P* = 0.002, respectively].

Antibiotic therapy of a duration >48 hours in well-appearing newborn infants for a presumed diagnosis of culture-negative sepsis or presumed sepsis (elevated CRP levels, but negative blood culture results) occurred more frequently during the baseline period [n = 10 (2%)] than during the QI-study period (n = 24 (1%), *P* = 0.06). It remained low during the surveillance period [n = 7 (1%)]. A high proportion of infants treated for culture-negative sepsis exhibited a mild elevation of CRP (<5 mg/dl) [baseline phase = 9/10 infants (90%), QI-study phase: 16/24 infants (66%) and surveillance phase: 4/7 infants (57%)].

**Fig. 3.**
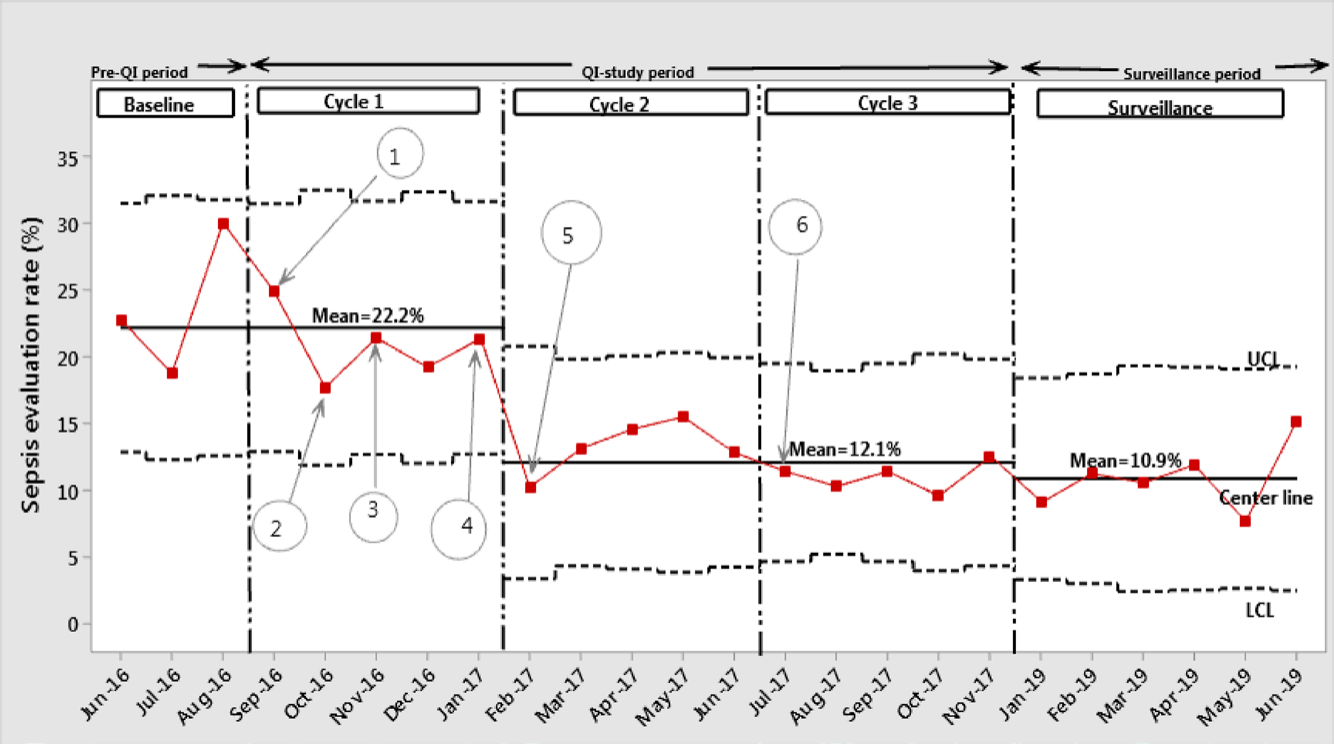
Annotated p-chart depicting monthly sepsis evaluation rate. Discription of annotations: 1: Education of residents and nurse practitioners on the use of web-based EOS calculator; 2: Nursing education; 3: Consensus on sepsis risk score cutoff levels; 4: Algorithm developed; 5: Implementation of EOS algorithm; 6: First revision of cutoff levels implemented.

**Fig. 4.**
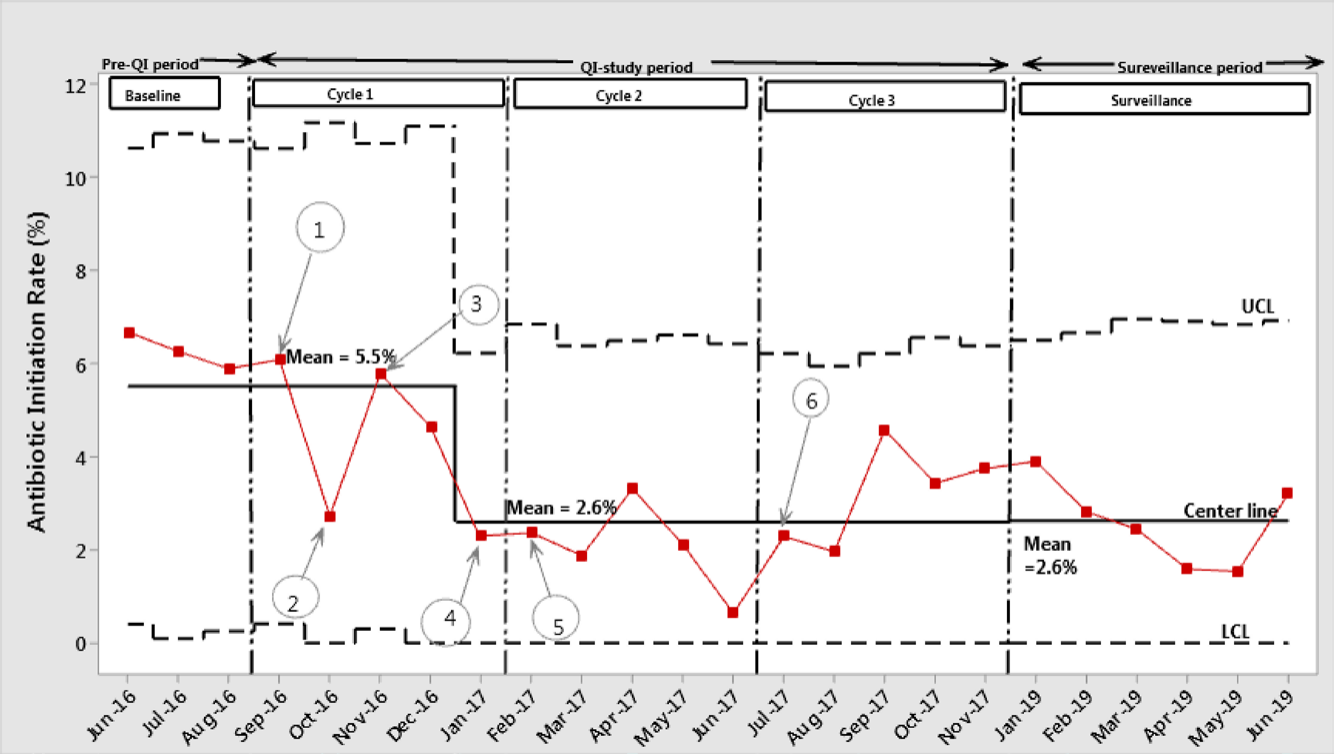
Annotated p-chart depicting monthly antibiotic initiation rate. Discription of annotations: 1: Education of residents and nurse practitioners on the use of web-based EOS calculator; 2: Nursing education; 3: Consensus on sepsis risk score cutoff levels; 4: Algorithm developed; 5: Implementation of EOS algorithm; 6: First revision of cutoff levels implemented (red circles indicate deviation rates above the target level after having implemented the EOS algorithm).

Both monthly SER and monthly AIR were plotted and analyzed using statistical process control p-charts (Figs. 3 and 4). For the monthly SER, after the special-cause signal shift at the end of PDSA cycle 1, we adjusted the centerline from the initial mean monthly SER of 22.2% to a new mean monthly SER of 12.2%. After the signal shift, the centerline and UCL and LCL were recalculated and plotted. For the rest of the study period, the process remained under control without any special cause variations resulting in increased monthly SER. Similarly, for the monthly AIR, the special cause-signal shift was demonstrated near the end of PDSA cycle 1, and the centerline, along with UCL and LCL, were recalculated and plotted. The centerline was adjusted to a new mean monthly AIR of 2.6% from the initial mean value of 5.5%. The process remained stable without special cause variations both during the study period. We noted no special cause process shift indicative of higher SER and AIR during the surveillance phase.

**Fig. 5.**
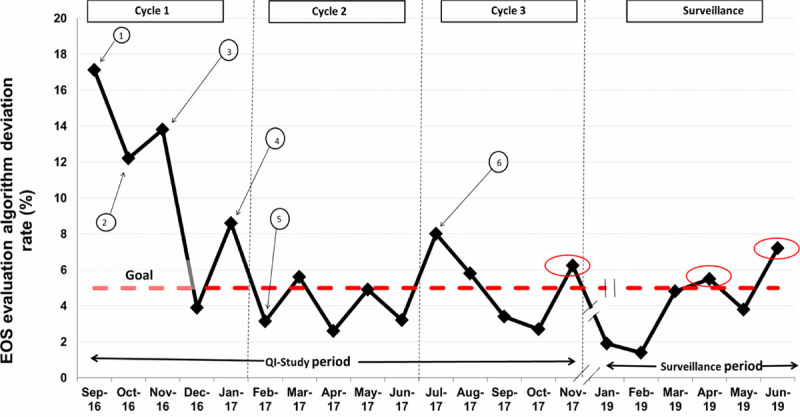
Process measure: annotated run chart depicting the monthly EOS algorithm deviation rates. * indicates corresponding cutoff value as per published guidelines of KPNC is <1; ≠ indicates corresponding cutoff value as per published guidelines of KPNC is 1–3; € indicates corresponding cutoff value as per published guidelines of KPNC is >3.

The monthly EOS algorithm deviation rate, the process measure, was monitored using a run chart (Fig. 5). The higher deviation rates above the intended goal of <5% were noted during the PDSA cycle 1 of the study before the implementation of the institutional algorithm. After the implementation, higher deviations were observed only during 3 months (area circled), mainly attributable due to variation in practice related to active maternal urinary tract infection. The 3 cases of culture-proven sepsis, the balancing measure, occurred during the study period (2 during the QI-study period and 1 during the surveillance period). Among all the culture-proven EOS cases, the SRS calculated after birth yielded values that were greater than the SRS cutoff vales for initiating the antibiotic therapy as per the institutional EOS algorithm. Hence, antibiotic therapy was initiated soon after birth without delay (Table 1). Similarly, even presumed sepsis cases were identified expeditiously during the hospital stay based on the abnormal CBC and/or elevated CRP levels.

**Table 1.**
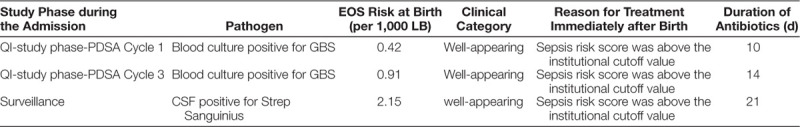
Balancing Measure: Culture-proven Sepsis during the Study Period

## DISCUSSION

In this single-center QI study, we demonstrated a successful implementation of an adapted web-based EOS calculator effectively reduced both SER and AIR without compromising the safety of all infants with culture-proven sepsis who were identified and treated timely. Despite the use of very conservative cutoff values of the SRS, we achieved significant reductions in both SER and AIR. Effective feedback to the first-line care providers when deviations from the guidance algorithm recommendations were observed (as monitored by the process measure) was the critical process that enabled the successful implementation of the institutional algorithm. We ascertained the safety profile for this new practice by monitoring both culture-negative and culture-proven sepsis rates. We collected the data on these measures through several PDSA cycles as well as during the surveillance phase.

The AAP new guidelines on EOS management^21^ among term and near term infants list 3 methods for EOS risk estimation: categorical stratification of risk, multivariate risk assessment using the web-based EOS risk calculator, and aggressive clinical observation method. Among them, the web-based application for estimating the EOS risk in neonates has gained broader acceptance across institutions, including our center, not only because it provides a reliable and objective EOS sepsis risk estimation, but is also a user-friendly tool with a definite recommendation for postnatal management of at-risk infants. The clear guidance of the EOS calculator and its flexibility to utilize extremely low sepsis score cutoff values were essential factors for generating trust in our institutional guidance algorithm and institutional improvement commitment.^22^ More importantly, our QI study revealed that our institutional sepsis risk among well-appearing neonates is approximately 3- to 4-fold higher than the institutional risk of KPNC. A high-risk nature of our population is probably attributable to several sociodemographic metrics, including teenage pregnancy rate,^23^ poor prenatal or late prenatal care, and lower socioeconomic status.^24^ These factors are associated with an increased risk of EOS. CDC 2016 national vital statistics showed higher rates of the indices mentioned above in Alabama compared to the national average.^25^ Hence, although we used the KPNC’s EOS incidence rate for computing SRS, our algorithm included SRS cutoff values that were 10-fold lower than the published recommendations of KPNC due to a high-risk nature of our population. In addition to the practice of obtaining blood culture,^26–28^ we also obtained ancillary testing such as CBC and serial CRP levels as biomarkers for EOS at our institution.^29^

The EOS risk estimates or SRS and the variables used for the SRS calculation were regularly incorporated in the electronic medical record during the entire study period. This process discouraged unjustifiable deviations from the guidance algorithm. Some physicians disregarded the protocol and initiated an evaluation of well-appearing infants when certain risk factors such as maternal urinary tract infection at the time of delivery or no prenatal care were present. Others have started antibiotics in infants born to mothers with chorioamnionitis or who had a brief febrile event. Monthly monitoring of the process measure enabled the team leaders to mitigate some of these barriers by providing monthly feedback with evidence-based resources to the first-line care providers. Moreover, at least once during a month, we conducted a discussion on EOS risk assessment and risk factors of EOS during the morning rounds. During these teaching sessions, we reinforced the utility and safety EOS risk calculator in combating the antibiotic overuse in the newborn nursery.

Although using the web-based EOS calculator, the providers need to be cautious. The computation of an SRS offers defined points of treatment. Still, the postnatal categorization into clinical illness or equivocal group based on the clinical assessment could result in falsely elevated EOS risk as the definitions of equivocal or symptomatic are vague and nonspecific. For instance, Akangire et al^30^ found that infants with small pneumothoraces and mild pulmonary hypertension were misclassified as symptomatic infants. Also, the threshold to intervene in symptomatic newborn infants may vary among practitioners.^8,13^ Thus, to simplify the decision process and enhance compliance, we only included well-appearing newborn infants. Another limitation is that the EOS calculator can still miss or delay the identification of EOS cases. In a high-risk cohort of neonates exposed to chorioamnionitis, 2 out of 5 infants who developed culture-positive EOS (one with *Escherichia coli*s sepsis and another with group B streptococcal sepsis), were well appearing at birth. One had reassuring SRS, indicating the need for only close observation, and the other one had scores indicating the need for blood culture and close observation. Both infants would have had a delayed initiation of antibiotics.^16^ Furthermore, in a **Table, Supplementary Digital Content,** http://links.lww.com/PQ9/A204, Kuzniewicz et al^14^ explicitly exhibited all infants from a large QI study who had a positive blood culture. They also included infants’ risk factors for sepsis and their SRS. Among these infants, 25 infants were well-appearing at birth. If we run hypothetical scenarios and apply various sepsis algorithms to these 25 infants, we would delay the diagnosis and antibiotic initiation in 22 infants using the EOS calculator algorithm and only in 9 infants if applying the CDC 2010 guidelines.

This study has several limitations. Being a single-center study is the major limitation of the current study as it lacks external validity. Although we never intended to create a generalized algorithm across institutions, our QI process was reliable and sustainable through multiple PDSA cycles, including the surveillance phase. A second limitation of the present study is the exclusion of all symptomatic infants from the study. Given the high-risk nature of our population with a higher incidence of EOS, a conservative strategy that involves staged incorporation of equivocal and clinical illness categories into the EOS guidance algorithm seemed a rational way to avoid deviation from the algorithm among practitioners. As mentioned in the Result section, few infants who received antibiotics had the diagnosis of culture-negative sepsis. Among them, unjustified antibiotics frequently occurred in infants who had a mild elevation in CRP levels and no other EOS indications. A failure to establish guidelines to manage minor elevations in CRP and monitor its trend was the third limitation of the current study. To overcome this limitation, we are currently standardizing a careful “watch and wait” strategy for clinically asymptomatic neoantes with mild elevation of CRP.

In conclusion, we describe a successful QI effort to safely reduce the number of blood tests and antibiotic usage in well-appearing newborn infants. These results were sustainable over an extended period. We highly recommend incorporating the neonatal EOS calculator in daily newborn nursery practice. However, the use of conservative SRS cutoff values may be essential for safety concerns, particularly in a high-risk population. This study marks a milestone in our efforts to improve the use of resources and limit unnecessary antibiotic use. We recognize that further interventions through multiple PDSA cycles are warranted to expand the population to include equivocal and clinical illness categories of neonates and gradually increase the SRS cutoff values to minimize unnecessary antibiotic therapy use further.

## DISCLOSURE

The authors have no financial interest to declare in relation to the content of this article.

## Supplementary Material


