# Supplementary material for: Implementation of a Modified Neonatal Early-onset Sepsis Calculator in Well-baby Nursery: a Quality Improvement Study
Source: Pediatr Qual Saf. 2020 Jul 7;5(4):e330. doi: 10.1097/pq9.0000000000000330 (PMC7351455; doi:10.1097/pq9.0000000000000330)
Supplement: Supplementary file 1 [file pqs-5-e330-s001.docx]

**Supplementary table: Baseline characteristics of the study population**

| **Phase** | **Baseline** | **PDSA cycle 1** | **PDSA cycle 2** | **PDSA cycle 3** | **Surveillance** |
| --- | --- | --- | --- | --- | --- |
| Time-period | June-16-Aug-16 | Sep-16-Jan-17 | Feb-17-June-17 | July-17-Nov-17 | Jan-19-Jun-19 |
| Total number of inborn infants, n | 542 | 907 | 797 | 918 | 889 |
| Excluded, n (%) | 32 (5.9) | 81 (8.9) | 61 (7.6) | 58 (6) | 89 (10) |
| Well-appearing infants, n | 510 | 826 | 736 | 860 | 800 |
| Gestational age, weeks (mean ±SD) | 38.4 ± 1.5 | 38.6±1.3 | 38.4±1.5 | 38.4±1.4 | 38.3 ± 1.5 |
| Birth weight, g (mean ± SD) | 3180 ± 508 | 3174 ±458 | 3198±471 | 3177±503 | 3166 ± 501 |
| Late preterm infants (34^0/7^ to 36^6/7^ weeks), n (%) | 61 (12) | 55 (7) | 75 (10) | 78 (9) | 95 (12) |
| **Well-appearing infants who underwent sepsis evaluation** | | | | | |
| Total number, n (%) | 122 (24) | 174 (21) | 98 (13) | 95 (11) | 87 (11) |
| Gestational age, weeks  (mean ± SD) | 37.7 ± 1.9 | 38.2±1.6 | 37.7 ± 1.9 | 38.1±1.8 | 37.7±2 |
| Birth weight, g (mean ± SD) | 3085 ±510 | 3086±512 | 3111 ± 564 | 3203 ± 551 | 3061±530 |
| Late preterm infants (34^0/7^ to 36^6/7^ weeks), n (%) | 33 (27) | 24 (14) | 25 (26) | 15 (19) | 25 (28) |
| Vaginal delivery, n (%) | 84 (69) | 124 (71) | 68 (69) | 70 (74) | 63 (72) |
| GBS positive, n (%) | 40 (33) | 44 (25) | 23 (23) | 21 (22) | 25 (29) |
| GBS negative, n (%) | 33 (29) | 71 (41) | 47 (48) | 52 (55) | 44 (51) |
| GBS unknown, n (%) | 49 (40) | 59 (34) | 28 (28) | 22 (23) | 18 (21) |
| Adequate GBS prophylaxis, n (%) | 26 (21) | 37 (21) | 33 (34) | 27 (28) | 31 (35) |
| Prolonged rupture of membranes (>18 h), n (%) | 16 (13) | 19 (11) | 12 (12) | 24 (25) | 24 (28) |
| Maternal fever (≥100.4 F), n (%) | 20 (16) | 28 (16) | 14 (14) | 20 (21) | 23 (26%) |
| Length of stay, days, median (IQR) | 3 (2, 4) | 3 (2, 4) | 3 (2, 4) | 3 (2, 4) | 3 (3, 5) |
| **Antibiotic therapy among the study population (well-appearing infants)** | | | | | |
| Initiation of antibiotic therapy for suspected EOS, n (%) | 32 (6) | 36 (4) | 15 (2) | 27 (3) | 21 (3) |
| Continuation of antibiotic therapy beyond 48h for culture-negative/probable sepsis, n (%) | 10 (2) | 10 (1) | 4 (0.5) | 10 (1) | 7 (1) |

Note: SD: standard deviation; GBS: Group B streptococcus; IQR: interquartile range
